# Bromide Calcium

**Published:** 1874-05

**Authors:** 


Bromide of Calcium.—This remedy, suggested by Dr. Hammond, has been investigated by Dr. Guttman, of Berlin, whose paper appears in the *Allgemeine Medicinische Central-zeitung*, December 6th. The latter finds it about one-third or one-fourth as strong as the bromide of potassium, and disagrees entirely both with Dr. Hammond's clinical and chemical theories of its value.

A physician, speaking of a patient who was prostrated by illness. remarked "he can hardly recover, since his constitution is all gone." "If his constitution is all gone," said a bystander," "I do not see how he lives at all." "O," responded the ready son of Esculapius, "he lives on the by-laws."—*Medical and Surgical Reporter.*

